# Stakeholder Recommendations to Increase the Accessibility of Online Health Information for Adults Experiencing Concussion Symptoms

**DOI:** 10.3389/fpubh.2020.557814

**Published:** 2021-01-11

**Authors:** M. Denise Beaton, Gabrielle Hadly, Shelina Babul

**Affiliations:** ^1^British Columbia Injury Research and Prevention Unit, British Columbia Children's Hospital Research Institute, Vancouver, BC, Canada; ^2^British Columbia Centre for Disease Control, Population and Public Health, Provincial Health Services Authority, Vancouver, BC, Canada; ^3^School of Population and Public Health, University of British Columbia, Vancouver, BC, Canada; ^4^Department of Paediatrics, University of British Columbia, Vancouver, BC, Canada

**Keywords:** concussion, mild traumatic brain injuries, accessibility, online resources, digital health, technology

## Abstract

**Background:** Concussion is a global public health problem. In Canada, concussion is among the top five reasons for workplace time-loss. Concussion results in physical, cognitive, and/or emotional symptoms that temporarily worsen with physical and mental exertion, such as viewing electronic screens. The Internet is the primary source of consumer health information. Studies on the end-user needs of adults with brain injuries in regards to digital health technologies largely focus on informational content. There is little to no research on the accessibility of screen-based informational websites and smartphone applications among this population.

**Objective:** The aim of this research was to involve stakeholders in the design of a comprehensive educational resource to guide concussion recognition, recovery, and return-to-work, called the Concussion Awareness Training Tool for Workers and Workplaces (CATT WW). In order to ensure both relevant content and appropriate delivery of the information to the target groups, participants were asked whether adaptations could increase the accessibility of online health information for the general adult population experiencing concussion symptoms.

**Methods:** Data have been generated through semi-structured in-depth interviews and focus groups with participants from across British Columbia (BC): workers from various industries who were in the concussion recovery process or had returned to work (*n* = 31); and healthcare or workplace professionals who support concussion diagnosis, recovery, and return-to-work (*n* = 16). Data were analyzed using NVivo 12. Before commencing data collection, ethical permission was granted by the University of British Columbia Research Ethics Board (H18-00604), and approval was received from WorkSafeBC Research Services.

**Results:** Participants (*n* = 47) recommended twenty adaptations or supplements to electronic screen-based digital health technologies.

**Conclusion:** Given the high prevalence of concussion among the working adult population, the symptom exacerbation commonly caused by prolonged use of electronic screens, and the demand for online educational resources, these findings can guide clinicians, researchers, technology developers, employers, and occupational health and safety committees to further support adults in concussion recovery and return-to-work.

## Introduction

Concussion, a term often used interchangeably with mild traumatic brain injury, represents 70–90% of all traumatic brain injuries and reportedly affects 100–600 people per 100,000 annually, depending on the definition criteria used (1, 2). In Ontario, Canada, recent analysis of linked data found an average annual incidence of 1,153 per 100,000 (3). These numbers are likely an underestimate of the true burden of concussion, given the lack of consistent reporting standards, misdiagnoses, and the inability to account for individuals who do not seek treatment (4, 5). The World Health Organization Neurotrauma Task Force defines mild traumatic brain injury as a blow or jolt to the head resulting in an acute disruption of brain function, manifested by a brief loss of consciousness (<30 min), confusion, or posttraumatic amnesia (<24 h) not accounted for by factors such as psychological trauma or alcohol/drug intoxication (6). A concussion can result in a variety of physical, cognitive, and emotional symptoms such as headache, blurry or double vision, anxiety, irritability, slowed reaction times, balance issues, and insomnia (7).

Most adults with a concussion recover within 1–3 months (8, 9). Expert consensus statements advise rest for 24–48 h, followed by gradually resuming normal activities in a step-wise approach, guided by the symptom exacerbation threshold (7, 10). Post-concussion syndrome, wherein symptoms persist past the standard recovery period, is estimated to occur in ~15–30% of individuals, with prevalence rates varying significantly depending on timing, measurement, and classification method used (11, 12). Any individual is susceptible to a concussion; however, the majority of research focuses on sport-related concussions, children and youth, and military populations, resulting in a dearth of resources to guide concussion recovery and return-to-work for the average adult (13, 14).

An online survey commissioned by the Public Health Agency of Canada found that 97% of respondents believed concussion was an important health problem, with 51% of respondents indicating that they knew where to seek information on concussion. However, 54% of respondents did not know how to respond to a potential concussion, and 60% reported being unable to recognize the symptoms of concussion (15). Given that concussion awareness often precludes diagnosis, and that the management of concussion recovery relies upon self-report of symptoms, increasing education and awareness among the general public is crucial. The primary method of accessing health information is online, with 94% of Canadian households and 87% of American households connected to the Internet (16–18). In 2013, the British Columbia (BC) Injury Research and Prevention Unit, in partnership with BC Children's Hospital Foundation, Child Health BC and the BC Ministry of Health, developed the Concussion Awareness Training Tool (CATT), an accredited online continuing education course for medical professionals. The course was then redeveloped in 2018, in partnership with the Public Health Agency of Canada via Parachute Canada, a national nonprofit dedicated to reducing preventable injuries. Redevelopments included updating and incorporating new and emerging evidence, as well as translating all modules to French. Since its debut in 2013, CATT has developed a series of free-of-charge online educational modules and resources for other audiences including parents, players, coaches, and school professionals. A module specific to intimate partner violence has now been included and one specific to high performance athletes is scheduled to launch in early 2021. The content in all modules is evidence-based and aligns with the 2017 recommendations from the consensus statement on concussion in sport and new and emerging evidence. The site is frequently updated to accurately reflect the rapid pace of evolving concussion research and related best practice (4).

Due to high demand and current international gaps in addressing non-sport related concussion among adults, CATT for Workers and Workplaces (CATT W&W) launched June 2019. CATT W&W provides easy access to current best practices for concussion recognition, diagnosis, treatment, and management tailored for workers and their families, workplaces including employers, members of safety associations, unions, and joint occupational health and safety committees. This is the first CATT resource addressing adults who are experiencing concussion symptoms as a key audience, and ensuring the accessibility of this information is critical. Digital health information is necessary in supporting individuals to make knowledgeable decisions about their care. The third most common Internet activity is searching for health information (19). Health information is abundant online, and sifting through resources increases demands on the user's memory (20). It is common for health information to be unsatisfying when it fails to consider individual needs (21). Through involving stakeholders in the design of the CATT W&W resource, the information compiled—and the methods of presenting that information—will be of greater relevance. The overall objective of this study was to create an innovative educational product that meets the needs of the end-users and increases concussion knowledge and awareness. The study intends to identify potential adaptations or supplements which can increase accessibility of digital health technologies.

## Methods

### Study Design

Among other aspects of the broader CATT W&W study on facilitators and barriers for recovery and return-to-work, the present study aims to identify adaptations and supplements to increase accessibility of online health information for the general adult population experiencing concussion symptoms. Qualitative research methods were used in order to generate knowledge grounded in human experience (22). We used interviews and focus groups to engage workers who had sustained a concussion and were in the recovery process or had returned to work, and workplace or healthcare professionals who support concussion recovery and return-to-work. The resulting data were assessed through qualitative inductive thematic analysis. Inductive thematic analysis allows for theoretical insights to be generated from data, as opposed to deductive research wherein theoretical hypotheses are tested via data collection (23). The ontology of this study fits within the social constructivist paradigm, recognizing that health does not exist separately from the person experiencing it (24).

### Recruitment

Participants were purposively selected with the following criterions for inclusion: Workers to be between 19 and 64 years of age, and to have sustained a concussion, either at the workplace or outside of work, and to be in the recovery process or to have recovered and returned to work; Healthcare and workplace professionals were required to be a member of a joint occupational health and safety committee, a workers' union, a WorkSafeBC (the provincial workers' compensation board) professional, or a healthcare professional who is involved in any/all stages of diagnosis, treatment, and management of concussions with the general adult population.

We chose to invite the participation of workers from diverse industries with high rates of concussion, in order to obtain rich and relevant data. Invitations to participate were sent via email to over 100 British Columbian occupational health and safety committees, unions, and organizations within industries with high rates of time-loss concussion claims: service sector; transportation and warehousing; trades; public sector; primary resources; construction; and manufacturing (25). Invitations to participate were disseminated via email among WorkSafeBC staff, and recruitment posters were displayed in common areas on bulletin boards in WorkSafeBC offices. Invitations to participate were disseminated via email among concussion clinics and healthcare professional associations. The invitation to participate letter and poster instructed interested parties to email or phone the researchers. Upon contact, potential participants were sent a consent form to review, and provided with the opportunity to ask questions regarding the study. Participants were offered the opportunity to take part in a one-on-one interview, or a focus group with other participants from the same industry.

### Participant Characteristics

Forty-six participants represented all health authority regions of BC, with approximately two thirds of workers residing in an urban location and one third in a rural location. One participant was from Ontario, and was forwarded invitation to participate from a contact in BC. The primary languages for workers were English (*n* = 30) and Senegalese (*n* = 1). The majority of workers experienced immediate symptom onset following the concussion-causing event (*n* = 30) while one experienced delayed symptom onset. The location of the most recent concussion-causing event was either at work (*n* = 14) or outside of work (*n* = 17), however, several participants in the latter category were commuting to or from work at the time of the concussion-causing event.

Ten female and six male healthcare and workplace professionals participated in the study. Occupations of workplace professionals were WorkSafeBC case managers (*n* = 2) and workers union advocate (*n* = 1). All healthcare professionals were employed at concussion clinics in the following occupations: occupational therapist (*n* = 5); kinesiologist (*n* = 2); psychologist (*n* = 1); physician (*n* = 1); neuropsychologist (*n* = 1); physiotherapist (*n* = 1); clinical counselor (*n* = 1); and office manager (*n* = 1).

### Data Collection

Data were collected from spring 2018—winter 2019 in Vancouver, BC. Sixteen participants chose to form three focus groups consisting of: three participants (workers; arts, entertainment, and recreation), three participants (workers; arts, entertainment, and recreation), and 10 participants (healthcare professionals; concussion clinic). The remaining 31 participants preferred one-on-one interviews (three healthcare professionals; three workplace professionals; 25 workers) (Tables 1, 2). Workers of those interviews, 13 were in-person, held at the BC Injury Research and Prevention Unit offices, and 18 were held by telephone for geographical reasons.

**Table 1:** Workers sample characteristics (*n* = 31).

**Category**	**Grouping**	***n***
Sex	Male	9
	Female	22
Age	25–35	10
	36–45	4
	46–55	13
	56–60	4
Industry	Health Care	15
	Arts, Entertainment and Recreation	7
	K-12 Education	2
	Scientific / Technical Services	3
	Construction	1
	Transportation and Warehousing	1
	Administrative and Support	1
	Security	1
Length of Time in Occupation	1–5 Years	5
	6–10 Years	7
	11–15 Years	10
	16–20 Years	3
	21+ Years	6

**Table 2:** Workers sample injury characteristics (*n* = 31).

**Category**	**Grouping**	***n***
Number of Concussions	1	15
	2	4
	3	7
	4	1
	5+	4
Mechanism of Injury^*^	Slip, Trip, or Fall	9
	Struck By/Against	15
	Car Crash/Collision	7
Level of Pre-Injury Concussion Knowledge^**^	A Lot	3
	Moderate Amount	6
	A Little	10
	None	11
Immediate Action After Injury^*^	Sought Medical Care	13
	Rested	4
	Resumed Activities	14
Family Support During Recovery^*^	Yes	19
	No	4
	Mixed	8

* Most recent concussion;

** Pre-injury concussion knowledge prior to first concussion.

In acknowledgement of their time, all participants received a $10 CAD honorarium in the form of a gift card to a coffee shop of their choice. Participants interviewed via telephone received an e-gift card. One participant requested that the researcher give their $10 CAD honorarium to a random person experiencing homelessness. While a noble gesture, this request was outside the parameters of the study and would not conform to the requisite paper trail of a purchase receipt and a confirmation of gift card form signed by the participant. With the participant's approval, the researcher instead donated the $10 CAD honorarium to a non-profit which serves Vancouver's youth population experiencing homelessness.

Two semi-structured question templates with key questions and discussion prompts were used. The question template for workers contained a brief section with sociodemographic questions (gender, age, primary language, current occupation, length of time in current occupational role), followed by a section on the experience of concussion including the mechanism of injury and diagnosis. The next section pertained to the experience of recovery and return-to-work. The final section referred to resources which provided information or support, ranging from resources used, and resources required but non-existent. The penultimate question was what participants wished workers and workplaces in their industry knew about concussion; this provided an opportunity for participants to summarize their message, their reason for partaking in this research, and for the researcher to member-check their responses to increase accuracy. The final question was whether there was a question the researcher should have asked, but did not. In response, the first worker participant interviewed proposed, “*how can online resources accommodate people currently experiencing concussion symptoms*?” This question was then added to the interview guides for all subsequent interviews and focus groups. At the conclusion of the interviews and focus groups with workers, participants were encouraged to email or call the researcher if they later remembered something they wished to say, and were offered a copy of the question guide. Six participants experiencing memory loss wrote follow-up emails to expound upon their responses in the interview or focus group, and these communications were included in the data analysis.

The question template for workplace and healthcare professionals was briefer than the question template for workers. Sociodemographic questions were restricted to gender and occupation, while background questions were the length of time in their current role, and how they are involved in supporting workers who sustain concussions. The workplace and healthcare professionals question template focused on resources—resources used in their work, gaps in resources for workers and families, the optimal delivery of resources, the added question of how online resources can be presented to accommodate people experiencing concussion symptoms—and once again ended with, “*Is there any question that I [the researcher] should have asked, but didn't*?” Throughout the interview and focus groups, the researcher member-checked, summarized what was discussed and asked if anything was missed. The interviews and focus groups for all participants ranged from 14 to 150 min long, and the average length was 45 min. The digital audio-recordings of interviews and focus groups were professionally transcribed.

### Data Analysis

Transcripts were cleaned of identifying information and the text was read through while listening to the audio to ensure accuracy. Raw data were archived with dates to provide an audit trail and a means of confirming our data analysis and interpretations for adequacy. An Excel spreadsheet was used to log all raw data, and to detail the progress in collecting and converting raw data to text that was subsequently analyzed by two independent researchers using NVivo 12, a qualitative analysis software. Step-by-step inductive thematic analysis was conducted in an iterative process, occurring concurrently with data collection. This allowed for newly emerging questions, such as digitally presenting information without provoking symptoms, to be explored. After familiarizing themselves with these data thoroughly, the researchers organized these data into a series of codes, described by Chapman, Hadfield and Chapman as “short statements that capture the meaning of the phrase, […] used to index the data and group together phrases with similar ideas or meaning” (2015, p. 203). The codes were then combined into themes, which were reviewed and compared for comprehensive coverage of the data, and checked against new raw data, until saturation—wherein no new themes emerge—was reached.

The advantages of thematic analysis were its usefulness to synthesize large amounts of information, examining the perspectives of diverse research participants, cataloging the similarities and differences, generating insight while producing a well-organized final report (26). The limitations of thematic analysis typically include the potential for researchers to let unconscious bias influence the results. In an effort to reduce implicit bias, researchers reflected on their own relationship with concussion; MDB conducted the interviews and subsequent analysis and had never experienced a concussion, and GH, who also conducted the analysis, had sustained several sport-related concussions. To demonstrate validity and minimize bias, researchers wrote memos justifying selection or rejection of particular phrases and codes, and met frequently for inter-rater comparisons and discussion.

### Ethical Considerations

This study was conducted in accordance with the Declaration of Helsinki (27). Before commencing data collection, ethical permission was granted by the University of British Columbia Research Ethics Board (H18-00604), and approval was received from WorkSafeBC Research Services. The participants' privacy was guaranteed by confidentiality throughout the entire study. Prior to the interview or focus group, each participant received a copy of the consent form via email with ample time to review. In the interview or focus group, participants were once again given the opportunity to examine the consent form and ask questions regarding the study. After being informed of the study aim, of their rights, and that participation was voluntary in all aspects, each participant provided their written consent, either in-person, via email, or fax. Each interview or focus group began only after the researcher reminded the participant(s) of the audio-recording. All names and places mentioned by participants have been changed to ensure anonymity; participants were given the opportunity to choose their own alias, or to have one assigned to them at random.

## Results

### Overview

Participants (*n* = 47) made recommendations for twenty different adaptations or supplements to digital health technologies in order to better provide online health information without provoking concussion symptoms, summarized in Table 3.

**Table 3:** Recommendations for adaptations or supplements to digital health technologies.

**Recommendation**	***n***
Provide audio options	17
Ensure easy navigation	16
Reduce visual stimulation	14
Ensure options to enlarge text	13
Provide color filter options	12
Use simple language	10
Accessible print options	9
Healthcare professional dissemination of printed materials	6
Feature personal stories of concussion recovery	6
Provide videos	5
Provide print resources on workplace location	4
Feature industry specific personal stories	4
Ensure a simple way to email information	3
Provide telehealth options	3
Develop mobile application	3
Utilize search term optimization	2
Prompted microbreaks	2
Geolocation targeted mobile ads	2
Auditory elements in low tones	1
Color coding	1

These recommendations address not only physical, cognitive, and emotional symptoms, but also opportunities to further increase education and awareness.

### Recommendations to Address Physical Symptoms

Participants reported that simply looking at an electronic screen can provoke headaches and/or nausea, while others attested that their vision was blurry, or they experienced sensitivity to light. Ensuring the availability of auditory options to convey health information was recommended by seventeen participants.

“*It would be the best for me personally, I guess, is kind of an audio option. But then also having something so you could kind of go back to it. But, yeah, I think in the beginning it was more like audio is sort of my best—the best way that I could get any information*.” [“Grace,” age 32, accountant, 1 concussion, on long-term disability leave].

“*The other thing I think specific to the population with brain injury is really about how you access that information. Because online is obviously the easiest and most accessible, but ironically the most difficult probably for people who have just had a concussion. I certainly couldn't engage on a screen for quite a long time. So I don't know, having different options like having an audio version or, yeah, some combination of, like, do you want to read it on a piece of paper, do you want to listen to it online or—not online, but do you want to just listen to it. Or do you want to watch the videos, so that maybe people throughout their concussion could have different ways of accessing it depending on what works best for them in the moment. Yeah, something like multimodal*.” [“Sarah,” age 33, occupational therapist, 1 concussion, full return to work].

Furthermore, several participants suggested that the auditory option be separated into sections by topic, so that those experiencing memory issues can choose to listen to specific sections (e.g., how to manage symptoms). Participants had suggestions on how to improve the visual components as well. Providing easy navigation was recommended by 16 participants.

“*I know as an educator, I can't quite remember, but something like if it's more than two or three clicks, it's too much*.” [“Deanna,” age 48, clinical nurse educator, 1 concussion, long-term disability leave].

“*But the less sort of— the least amount of click-on this, click-on this, register this, click-on this, user password, no password, is to burden someone. On my brain anyway. That's what I really found*. [*Cause I think people, women in particular around my age, aren't really great at the computer anyway. And then when you have to dig really deep into—before you get to the starting page like it should open to a website or something and give a good little table of contents*]”. [“Amy,” 59, speech language pathologist, 1 concussion, full return to work].

Fourteen participants suggested reducing visual stimulation on the website, which included subdued colors (e.g., navy instead of black), avoiding fast moving videos, and having a minimal amount of information presented per page.

“*Not flashy stuff. Something that's flashing across the, I mean, any—I found, you know, when I first sustained it I could—less than a minute I could spend on a site—like on the computer, 30 seconds maybe till I really felt symptoms. Well, I felt symptoms all the time but—till they felt even worse*.” [“Victoria,” age 54, registered nurse, 1 concussion, full return to work].

Thirteen participants recommended providing options to enlarge text size, with the caveat that it should not increase the need to scroll on the page.

“*But even now my vision isn't great, so being able to enlarge it. I have a—like on my computer I can do that but then you lose part of the page. You lose part of the text so then you—you can't—it's hard—you have to slide the whole thing back and forth to make it big enough, but then I can't see the whole page, the whole width of the text. Which is not good when you have, like, nausea*.” [“Mayra,” age 45, teacher, 2 concussions, partial return to work].

“*Clear, larger font. There were some fonts even that I use at work that I realized I was looking at them and all of a sudden it was just all blurry*.” [“Charlotte,” age 50, registered pharmacy technician, 2 concussions, full return to work].

Twelve participants recommended the provision of color filter options, so end users could adjust the screen to their preferences. Nine participants recommended ensuring an easily visible option to print resources, or to provide print resources mailed to the end-user following entry of an address.

“*Reading the screen, obviously, is hard and being able to read something on paper is great. But probably just the first week or two that would be the most important*.” [“Raymond,” 34, stunt performer, 3 concussions, full return to work with adaptations].

One participant provided advice regarding the type of auditory elements:

“*If there is going to be any type of sound, that it's a very low calming sound, nothing sharp*.” [“Charlotte,” age 50, registered pharmacy technician, 2 concussions, full return to work].

### Recommendations to Address Cognitive Symptoms

Participants experienced a loss of concentration and memory while recovering from their concussion and provided recommendations of potential adaptations to address these cognitive symptoms. Ten participants recommended using simple language.

“*Like keeping it very simple for the language. Easy to understand*.” [“Justine,” age 26, communications advisor, 1 concussion, full return to work].

Furthermore, five participants recommended providing educational videos to sustain interest, and present content in a memorable way.

“*I'm finding for people who have an actual concussion, reading, challenging. So maybe videos. Like, here's a video tutorial on concussions and what you can expect. As opposed to, here's a 200-slide PowerPoint with very detailed small font and lots of graphics that'll give me a headache. And I'll get through three or four slides, yeah. Not going to happen for a person with a head injury*.” [“Bruce,” workers' union advocate].

“*People watch TEDx or inspirational videos or will listen to things. So if it was engage—like, it'd have to maybe be for those ones that are, tend to be more passive, if it was really engaging, that they would then want to, and it would also help their memory if it was something that was more exciting*.” [“Penelope,” occupational therapist].

Two participants suggested prompted microbreaks, so that end users with concussion symptoms can rest.

“*I think it's a lot of the public in general, but sometimes people think that they can just push through it* [experiencing symptoms] *and they'll feel better. They'll push through it and it'll be okay. And so that's when the breaks become important*.” [“Janelle,” kinesiologist].

One participant suggested that information sheets and sections of the website be color coded, to denote which resources are for which audience, eliminating the mental strain of searching for relevant information.

### Recommendations to Address Emotional Symptoms

Participants stated that the experience of emotional symptoms, such as anxiety, depression, and mood swings, was unexpected. Physical and cognitive symptoms are well reported in media reports of professional athletes sustaining and recovering from a concussion. The emotional symptoms that can occur during concussion and post-concussion syndrome recovery were previously unknown to participants and their families, and they reported receiving little information or support to cope. Participants suggested two things that could help manage emotional symptoms: six participants recommended featuring personal stories of individuals recovering from emotional symptoms of concussion and post-concussion syndrome, to help normalize and destigmatize such experiences, and three participants in rural locations recommended the provision of telehealth options to connect with mental health experts that they otherwise would not have access to.

“*There was a couple of times where I was, like, is this really in my head, or am I really experiencing this? And even now somewhere along the way I have to do a real good check-in with myself saying, you know, am I really making this more than it should be? Like, I'm not quite understanding—I shouldn't be feeling like this anymore. So it was that second guessing on my part. So definitely a section in there for people to refer to, to say, actually yeah, those people experienced something similar. So a bit of a reference to say, actually no, it's not in my head. Because then you go down a different path of oh, I'm losing it*.” [“Charlotte,” age 50, registered pharmacy technician, 2 concussions, full return to work].

“*I mean, it would be nice to have access to someone who's an expert in it. I was reluctant to go to like a—we have a chiropractor in town that calls himself an expert in concussions. And I didn't feel comfortable with that. And there were no other experts unless I left town to go to. And it would be nice to have telehealth support from an expert in the field so you're getting the best up to date information at the time. Cause I think that the physicians, one, they don't have time and, two, they don't— they may not all be up to speed on the current recommendations. It's not super helpful. And just—and having to go in and see them, just for no—other than, you know, they helped me get back to work, it just felt like a real effort and with no—nothing. I got nothing out of it other than get my note for work. […] rather than trying to Google and find, you know, anecdotal stuff that—and then you think, well, it's just, you know, is this just for this person or—yeah*.” [“Victoria,” age 54, registered nurse, 1 concussion, full return to work].

### Recommendations to Increase Education and Awareness

Over half of participants did not immediately seek medical help following their most recent concussion-causing event, and the majority of those that did pursue immediate care were guided by their experience of previous concussion(s). Participants made five recommendations to harness the potential of digital health information resources to increase concussion education and awareness. Six participants suggested supplying print resources featuring the CATT W&W website address to healthcare providers so that adults diagnosed with concussion will receive timely information.

“*So again, you'd want the physicians to be able to give those resources to their patient when they come in. It'd be ideal if somehow the medical association was included. Like, hey, by the way, we're developing this tool. If you ever get a patient who has a head injury that's work related or not, here's the tool and the resources are being developed. Please refer your patient to that site. Really important. [Cause otherwise the patient walks out of their 8-min consultation with the average physician and goes, what do I do now? My specialist I'll see in 3, 4, 5, 6 months. I can look online. I can Google it, and I'll be overwhelmed by all the information. I don't know what's legit, what's not, what's current, what's not. I think one portal, one doorway. Here's where you go if you've had a head injury and then go from there]*”. [“Bruce,” workers' union advocate].

Four participants recommended the provision of print resources on location in industries where occupations do not involve computer use and/or reporting of a concussion may be stigmatized.

“*Yeah, just—I think if the information is easy to get, then people will be more likely to pick it up. Even if somebody's a little bit paranoid they still think, oh, people are going to think I'm a wuss but at the same time my head's really starting to bother me. The brochure's right there, I'll just stuff it in my bag when nobody's looking. And then they'll go home and read it*.” [“Adrian,” age 50, professional wrestler/stunt performer, 5+ concussions, full return to work].

“*Even if you give out, like, a pamphlet that won't hurt either, right. They could read on their own time and get them to be aware of it*.” [“Carlos,” age 43, construction worker, 3 concussions, on unpaid leave].

Four participants recommended that personal stories of concussion recovery, return-to-work, and prevention be separated by industry in order to be more relatable.

“*And I think even it would be important to have it from various walks of life, right, have a healthcare person. [Cause healthcare speaks to healthcare. That would be their peer, right. Construction, for example, I mean, we have some pretty serious injuries that come out of construction. Just let's have a wide range of people who have different occupations and different age groups too. Because I think then people can—it's more relatable if they feel like it could be captive in their own voice. I think that they—you probably are going to capture a bigger audience that way]*”. [“Dolores,” case manager, WorkSafeBC].

Three participants suggested a button on the website which made it easy to email information to a family member or colleague. Three participants suggested developing a mobile app, or ensuring that websites are mobile friendly. Two participants recommended using geolocation capabilities to target informational mobile ads for specific industries, e.g., for the film industry, at the site of major studios.

“*So potentially we could do something around somebody walks onto a lot and an ad pops up and, you know, it's just a little prompt about concussion or about injury prevention*.” [“Raymond,” 34, stunt performer, 3 concussions, full return to work with adaptations].

Two participants suggested that search term optimization be broadened to promote concussion health information websites among the results following Internet users searching associated terms such as, “whiplash,” “car crash,” as well as common concussion symptoms.

“*It would be wonderful if you could—if anybody Googled* “*headaches*” *if it could come up. I guess maybe if I had Googled, like, headaches and was able to link what I was feeling and seeing—might have instigated me to go to the doctor maybe quicker. Rather than waiting. It's, like, no, this is not right, this isn't just whiplash. There's no relation to the neck what you're experiencing*.” [“Carol,” 59, scheduler, 2 concussions, partial return to work].

The workplace and healthcare professionals who participated in the study provided recommendations based on an agglomeration of experience supporting adults with concussion, and felt frustrated that they lacked quality resources to recommend to patients or clients. Participants who had sustained concussion(s) felt distress due to insufficient information. This often delayed their choice to seek diagnosis, resulted in delayed recovery and return-to-work, and greatly impacted their care decisions as well as their physical, mental, and social well-being.

### Principal Findings

The present study is a component of broader research on the experiences of concussion diagnosis, recovery, and return-to-work among the general adult population. These findings informed the creation of the CATT W&W e-learning course and related resources. Aligned with the current research literature, the initial aim of the CATT W&W study pertained solely to the needs of end-users for informational content and ensuring the credibility of that health information. Throughout the course of the study, participants emphasized the requirement for the presentation of online health information to be adapted or supplemented in order to increase accessibility for adults experiencing concussion symptoms. Participants made 20 recommendations to address physical symptoms (auditory options; easy navigation; fewer visually stimulating elements; enlarged text; color filter options; easily receiving printed resources at home, through self-printing or mail; and pleasant auditory sounds), cognitive symptoms (use of simple language; educational videos; prompted microbreaks; color coded information), emotional symptoms (personal stories; telehealth options), and to increase concussion education and awareness (print resources provided to healthcare providers; print resources for specific industries; industry specific personal stories; easy email options to disseminate information; mobile apps; utilizing geolocation to target ads; and search term optimization).

The categories these recommendations fall in are permeable and reflect the intended context of the participants' suggestions. Providing an easily visible option to print resources at home, or providing print resources mailed to the end-user following entry of an address, was suggested by nine participants as a solution to physical symptoms exacerbated by screen time. The same suggestion could be made to address cognitive symptoms, as a physical copy of the Return-to-Work Strategy, for example, could provide a reminder of the stepwise approach to recovery for those experiencing memory loss after they have left the online website. Or, it could fall under increasing education and awareness, as the same resource could be posted in the workplace or home of the individual recovering from a concussion, where it would be viewed by colleagues or family and friends, providing a visible reminder for an invisible injury. Occasionally, participants' recommendations contradicted one another; to address emotional symptoms, several participants suggested providing personal stories, as they would take comfort in knowing they were not unique in their experience. Other participants felt overwhelmed with the amount of anecdotal evidence found online. They would prefer access to expert opinion via telemedicine, to receive tailored advice. Given that no two concussions are alike, it is not surprising that recommendations varied, and reveals the need for a broad array of adaptations and supplements to increase the accessibility of online health technologies and support concussion recovery.

Including all twenty of these recommendations into the development of CATT W&W was beyond the initial scope of the project, however, certain suggestions aligned with online public health information best practice previously adhered to by CATT (simple language, easy navigation, pleasant auditory sounds, educational, and engaging videos). For the provision of print resources to specific industries and healthcare providers, all CATT print resources are available for order at low, cost-recovery prices. Other recommendations were easily incorporated *ad hoc*, such as providing print and auditory options. All modules of the CATT W&W e-learning course are available for download either as plain text, or as audio files. The 20 recommendations reveal a gap between participants' needs and what the healthcare system provided, and can guide the provision of future accessible concussion digital health information for the general adult population.

### Comparison With Previous Work

This is the first study to date which considers potential adaptations and supplements to increase the accessibility of online health information in support of concussion recovery and return-to-work. Managing concussion requires consistent symptom monitoring which informs adaptations to the pace of activity resumption. Until recently, expert consensus statements recommended that concussion recovery necessitated complete rest until total symptom resolution. Individuals recovering from a concussion were advised to avoid physical activity that elevated their heart rate, tasks which increased the cognitive load (e.g., work or school), and exposure to sensory stimuli (light, noise, screens). A growing body of recent evidence suggests that resuming usual activities after 24–48 h of rest may lead to a faster recovery provided the individual does not exacerbate symptoms (8). Given that medical health professionals are not uniformly aware of the updated recommendations for concussion recovery, online health information plays a critical role in informing care.

A concern among medical professionals and workers' compensation boards is discerning “true” concussions from scenarios where an individual is “intentionally fabricating or exaggerating symptoms to achieve an external goal (e.g., malingering) or otherwise fabricating or exaggerating symptoms (i.e., feigning and dissimulating) to obtain attention from medical professionals or to avoid work, school, military service, or other responsibilities” [(28), pg. 96]. Malingerers may seek online health information to bolster their performance of symptoms, though studies have found that those who malinger can be identified by intentional poor performance on cognition tests, with those with mild TBI often exhibiting poorer effort and worse cognitive performance than those with moderate or severe TBI (29). New methods are being developed to discriminate fake from true brain injury, such as using latency of left frontal neural responses during old/new memory recognition (30). Regardless of the minority of malingering individuals, providing adaptations to increase the accessibility of online public health information benefits those experiencing concussion symptoms, as well as Internet users with varying visual, mobility, hearing, and intellectual disabilities (31).

Research on the potential of harnessing technology for concussion care includes virtual reality gaming as an assessment and/or neurorehabilitation tool (32, 33); eye tracking technology for concussion assessment among sports medicine clinicians (34); mobile applications (35); wearable technology, such as helmets and mouthguards, to aid in the detection of sports-related concussion (36); blood-based biomarkers (37); fluid biomarkers and genetic testing (38) and more. While the healthcare and workplace professionals interviewed within the present research acknowledged the burgeoning field of technological research and development in regards to concussion care, they stressed the need for low-tech solutions. Namely, evidence-supported, accessible resources which they can use to support workers with concussion in their recovery and return-to-work. Inappropriate communication which is limited to a brief exchange within a doctor's office, an emergency department, or a phone call with a workers' compensation board case manager, may result in poor understanding and difficulty to recall the discharge care or return-to-work plan (39).

In the early phases of recovery from concussion, research suggests that individuals may experience difficulty satisfying the basic psychological needs of autonomy, competence and relatedness, and early interventions offered should be attentive to the potential emotional symptoms which can arise (40). Chang et al. (41) found that work-related mild traumatic brain injury often resulted in long term consequences, including challenges in daily activities and return-to-work. Workers experiencing mild traumatic brain injury are 3.5 times more likely than the general unemployed population to remain unemployed one year later (42). Workers in positions of precarious employment, such as part-time, temporary, short or fixed-term contract, self-employed or seasonal work comprise 30–32% of the Canadian workforce. The lack of security, low control over work processes, as well as social and economic vulnerability experienced by precariously employed workers is compounded by illness or injury (43). Though the challenges are clear, the opportunity exists for organizations and researchers to champion a public health and prevention approach to safeguard employees' health (44).

### Limitations and Future Directions

Findings from the present study are instructive for clinicians, researchers, technology developers, employers, and occupational health and safety committees to recognize the barriers and facilitators for individuals with concussion symptoms in accessing online health information. Given the recent shift toward working and studying from home, these findings could also inform organizational strategies to enable individuals recovering from concussion and post-concussion symptoms in accessing resources online. Strengths of the study include soliciting the perspectives of workers who sustained a concussion and returned to work or were in the recovery process from a wide array of industries, as well as the healthcare and workplace professionals who support these processes. The recruitment methods resulted in a sample which is predominantly English-speaking, securely employed, middle-aged, and female, which may negatively impact the transferability of results. These findings could provide a fuller picture by including the perspectives of the family members and colleagues of individuals recovering from concussion, as well as a more representative sample of the general population, including precariously employed workers, self-employed workers, older workers (aged 65 years and above), young workers (aged 25 years and below), and recent immigrant populations.

However, while the results cannot be generalized to everyone experiencing concussion recovery and return-to-work, they can be used in developing adaptations and supplements to the presentation of online health information for concussion, given the similarity of responses. Researchers may wish to explore a longitudinal investigation of the adaptations and supplements required by individuals recovering from a concussion at the various stages of recovery to ascertain which are most beneficial at certain time points. Research is also needed as to the uptake of health information to increase concussion awareness and education by various industries. Within our results, there appears to be a relationship between traditionally masculine occupations and the stigmatization of concussion symptoms, particularly in regards to emotional symptoms, as well as the disparate requirements of rural and urban populations; more research is needed to investigate the influence of these variables on experiences of concussion recovery and return-to-work.

## Conclusions

Participants shared the adaptations and supplements which may have enabled them to receive the right online health information at the right time, further supporting concussion recovery and return-to-work. The diagnosis, evaluation, management, and determination of recovery from concussion relies largely on clinical assessment. Currently there is no sole instrument sufficient to assess concussion in isolation, and research supports comprehensive, multidomain assessment approaches to concussion, and a multi-disciplinary approach to care for post-concussion symptoms (45). Research suggests that a one-size-fits-all method of concussion care is ineffective (46). Every concussion is unique, but the way in which the general adult population seeks health information is not. Searching for online health information is one of the foremost uses of the Internet, and may preclude the decision for an individual to seek medical care. Harnessing the potential of online health information to increase education and awareness about concussion requires an array of adaptations and supplements to accommodate not only symptoms, but also the nature of the work being returned to, and the context in which the individual is recovering.

## Data Availability Statement

The datasets presented in this article are not readily available because informed consent was not obtained from participants to facilitate data sharing and scholarly reuse; therefore, these qualitative data cannot be legally and ethically shared. Requests to access the datasets should be directed to M. Denise Beaton, denise.beaton@bccdc.ca.

## Ethics Statement

The studies involving human participants were reviewed and approved by The University of British Columbia Research Ethics Board. The patients/participants provided their written informed consent to participate in this study.

## Author Contributions

SB was the Principal Investigator and encouraged MDB to carry out the research and supervised the analysis and findings of this work. MDB wrote the manuscript with input from SB and GH. GH analyzed the data, and worked with MDB to establish inter-rater reliability. All authors reviewed the manuscript and provided final approval. All authors contributed to the article and approved the submitted version.

## Conflict of Interest

The authors declare that the research was conducted in the absence of any commercial or financial relationships that could be construed as a potential conflict of interest.
